# Design of an Adaptive Human-Machine System Based on Dynamical Pattern Recognition of Cognitive Task-Load

**DOI:** 10.3389/fnins.2017.00129

**Published:** 2017-03-17

**Authors:** Jianhua Zhang, Zhong Yin, Rubin Wang

**Affiliations:** ^1^Intelligent Systems Group, School of Information Science and Engineering, East China University of Science and TechnologyShanghai, China; ^2^School of Optical-Electrical and Computer Engineering, University of Shanghai for Science and TechnologyShanghai, China; ^3^Department of Mathematics, Institute of Cognitive Neurodynamics, School of Science, East China University of Science and TechnologyShanghai, China

**Keywords:** adaptive functional allocation, cognitive task-load, electrophysiology, dynamic pattern recognition, man-machine system

## Abstract

This paper developed a cognitive task-load (CTL) classification algorithm and allocation strategy to sustain the optimal operator CTL levels over time in safety-critical human-machine integrated systems. An adaptive human-machine system is designed based on a non-linear dynamic CTL classifier, which maps a set of electroencephalogram (EEG) and electrocardiogram (ECG) related features to a few CTL classes. The least-squares support vector machine (LSSVM) is used as dynamic pattern classifier. A series of electrophysiological and performance data acquisition experiments were performed on seven volunteer participants under a simulated process control task environment. The participant-specific dynamic LSSVM model is constructed to classify the instantaneous CTL into five classes at each time instant. The initial feature set, comprising 56 EEG and ECG related features, is reduced to a set of 12 salient features (including 11 EEG-related features) by using the locality preserving projection (LPP) technique. An overall correct classification rate of about 80% is achieved for the 5-class CTL classification problem. Then the predicted CTL is used to adaptively allocate the number of process control tasks between operator and computer-based controller. Simulation results showed that the overall performance of the human-machine system can be improved by using the adaptive automation strategy proposed.

## Introduction

In safety-critical human-machine integrated systems, human operator, and machine are integrated collaboratively to accomplish complex tasks, in which the operator has to adapt to unforeseen disturbances or even system failures under dynamic process task environment. In such fields as public transportation (Yang et al., 2009; Khushaba et al., 2011) aeronautics and astronautics (Sauvet et al., 2014) and nuclear engineering (Bobko et al., 1998), catastrophic accidents may occur due to operator performance breakdown. However, at the current stage of technological development the complete removal of humanistic supervision and/or intervention from the human-machine systems arising in those fields is still impractical. Thus, researchers started to explore how to detect the risky operator functional state (OFS), which may fluctuate over time. In the framework of OFS analysis (Hockey et al., 2003), human cognitive task-load (CTL) can be defined as the portion of information processing capacity of the operator required to meet the performance requirements of the human-machine system (Eggemeier et al., 1991). The CTL can be regarded as the mental workload (MWL) of operators while performing cognitive tasks. In other words, the terms of CTL in this context and MWL assessed under cognitive tasks are somehow interchangeable, but the former is more suited to describe operator mental stress under complex human-machine cooperative task requirement in the framework of OFS analysis (Byrne and Parasuraman, 1996; Parasuraman and Riley, 1997; Borghini et al., 2014; Lupu et al., 2014).

Traditional CTL assessment methods mainly include subjective ratings (Hart and Staveland, 1988) and task performance measures (Grant et al., 2013). However, these two techniques may either interrupt the current task execution or impose secondary tasks on the operator. An alternative method is to use physiological measures, which is featured by continuous-time, non-invasive, and objective assessment (Craven et al., 2015). Since the physiological signals provide real-time information about the cognitive mechanism underlying performance variations, the variations in physiological features can be measured before manifest performance decline. In this regard, electroencephalogram (EEG) has been widely used by researchers in the emerging cross-disciplinary area of neuroergonomics to quantify CTL variation since it can reflect the change of neural activity in central nervous system (Zhu et al., 2014). Although EEG still has limitations in 3-D localization of neural activities (Peng et al., 2013), its advantages include high temporal resolution and readiness of signal measurement. The continuous and long-duration EEG measurement under human-machine task environment is essential for CTL assessment. It was shown that CTL can be classified into a few discrete levels based on the temporal variation in power spectral density (PSD) of EEG rhythms in certain frequency bands such as delta (0.5–4 Hz), theta (4–7 Hz), alpha (8–13 Hz), beta (13–25 Hz), and gamma beta (26–33 Hz; Gundel and Wilson, 1992; Sun and Yu, 2014). Moreover, CTL can also be assessed by using electrocardiogram (ECG) signal which reflects the activity of autonomous nervous system. In particular, ECG indices such as heart rate (HR) and heart rate variability (HRV; Mulder et al., 2004) are usually used, along with various EEG features, to characterize the CTL. The hybridization and fusion of EEG and ECG features can overcome the inadequacy of the features extracted from a single source signal (Yang and Zhang, 2013). The combination of the two measures helps us have a better understanding of the effect of CTL levels on neurophysiological activities.

The ultimate goal of CTL assessment is to design and implement an adaptive automation (AA) system, which is capable of regulating the momentary CTL to optimal level and thus maintaining optimal performance of human-machine system. AA allows for reallocation of type and amount of cognitive tasks between human operator and the machine. Since inappropriate intervention may disrupt the normal operation of the system (Scerbo, 1996), accurate CTL assessment is the prerequisite for the design and implementation of AA systems (Kaber et al., 2006). The early work by Pope et al. showed that a “biocybernetic” system can improve the engagement of the operator in Multiple-Attribute Task (MAT; Pope et al., 1995). The engagement index (EI), defined as the ratio between beta power and the sum of alpha and theta power related to certain EEG measurement channels, was used to indicate how much the operator concentrates on the tasks. It was shown that negative feedback leads to a stable short-cycle oscillatory EI index, which indicated that the operator was stably involved in the tasks. Parasuraman et al. also performed experiments under MAT task on 27 volunteering participants, which were divided into three groups, “model-based adaptive control,” “performance-based adaptive control,” and “non-adaptive control” (Parasuraman et al., 1996). Statistical analysis results showed that the performance of participants in two adaptive control groups was significantly higher than that in non-adaptive control group. The work of Freeman et al. (1999) and Prinzel et al. (2000) indicated that AA has a positive influence on CTL based on NASA Task Load Index (TLX). Haarmann et al. implemented AA strategy in a simulated flight task and found that if the AA system reallocated tasks frequently the CTL may increase, in other words the frequency of task allocation may affect the stability of the AA system (Haarmann et al., 2009).

This work first examines how the EEG and ECG features are correlated to task performance. An automation-enhanced Cabin Air Management System (aCAMS; Lorenz et al., 2002; Manzey et al., 2008) was used to simulate a complex and safety-critical human-machine process control system. Computational intelligence techniques, such as support vector machine (Yang and Zhang, 2013), fuzzy inference system (Zhang et al., 2013), and artificial neural network (Russell and Wilson, 2001), has been used for automatic CTL classification. Specifically, SVM approach is based on the principle of structural risk minimization (Vapnik, 2000) and thus suitable to deal with high-dimensional physiological features. In literature, static CTL classifiers, such as feedforward ANN (Russell and Wilson, 2001) and adaptive-network-based fuzzy inference system (Zhang et al., 2013), were designed to identify temporal variations in CTL. In static classifier, the current CTL level solely depends on the current physiological features. Nevertheless, since the variations in brain and cardiac functional state are continuous, the current CTL level may be also correlated with the historical (past) data at the previous time steps. Taking into account the above issue, we used least square support vector machines (LSSVMs; Suykens and Vandewalle, 1999) to design a dynamic pattern classifier based on Non-linear AutoRegressive model with eXogenous (NARX) inputs. In Zhang et al. (2013), the CTL level was predicted by using fuzzy inference system model with only two EEG-related input features, which are the ratio between the EEG theta and alpha spectral power. However, the use of only two input variables for the fuzzy model cannot estimate the multiclass CTL accurately enough. A higher false alarm rate resulted from the use of a lower-dimensional model with excessively parsimonious structure. Moreover, in Zhang et al. (2015), an adaptive SVM classifier was built based on boundary support vector machines in order to improve the CTL classification accuracy in the case of non-stationary EEG and ECG features. However, the proposed A-BSVM method has to re-compute multiclass support vectors at each time step and is thus computationally inefficient. A dynamical learning set, constructed by preserving those support vectors that are correctly classified, was used to retrain the BSVM classifier. In this work, the dynamical learning set based paradigm is replaced by a NARX dynamic model with output feedback (recurrent) mechanism. In comparison with our previous work, the current work used two different NARX LSSVM models and more EEG features to enhance the CTL classification performance. In particular, the designed CTL classifier is validated on a simulated adaptive HM system.

The rest of this paper is organized as follows. In Section Experiments and Data Preprocessing, the data acquisition experiments and data preprocessing approach are described. Section Dynamic CTL Classifier Based on Physiological Features describes the methods of target class determination, feature reduction and smoothing, and dynamical CTL classification. The design and simulation of an adaptive human-machine system are presented in Section Design and Simulation of Adaptive Human-Machine System. Section Discussions analyzes and discusses the CTL classification and adaptive automation simulation results obtained. Finally, Section Summary and Conclusion concludes the paper.

## Experiments and data preprocessing

### Experimental setup

#### Experimental tasks

The experimental purpose is to collect physiological and performance data of human operator under simulated process control tasks provided by aCAMS, which was used to simulate monitoring and control of the air quality in a space capsule. The measured data is then used for participant-specific CTL model construction and AA system simulation.

**Figure 1:**
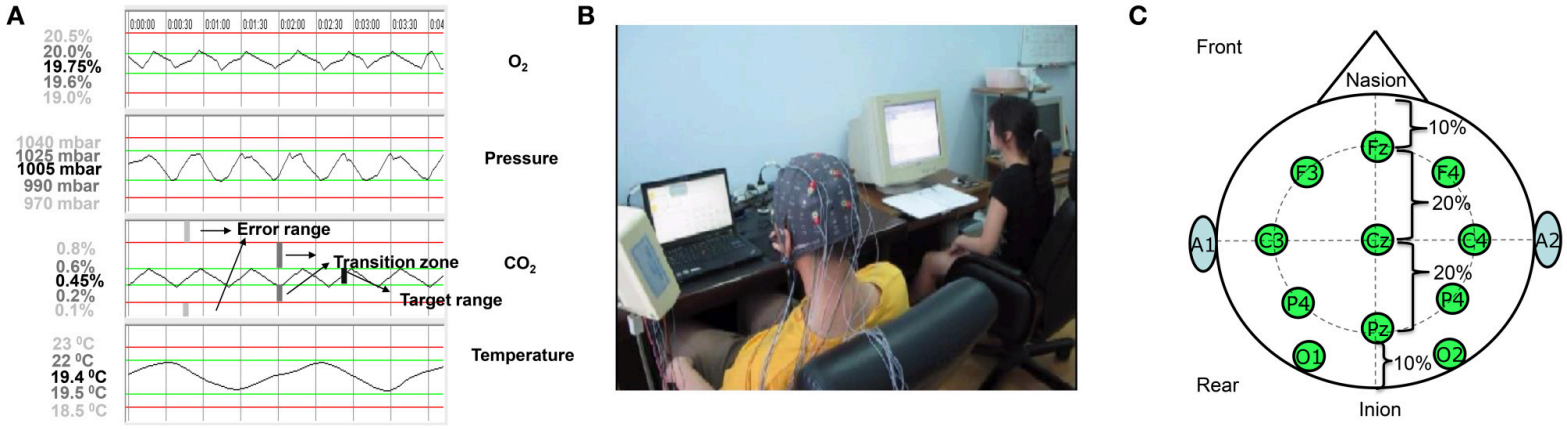
**Human-machine cooperative task environment: (A)** The four output time responses of the aCAMS; **(B)** A snapshot of a volunteer participant engaged with the aCAMS while his psychophysiological responses are being measured under lab setting; **(C)** EEG measurement electrodes selected. The output response of each of four subsystems is shown top down. For each subsystem, the interval between two green lines defines the target range. The interval between the upper red and green lines and the interval between the lower red and green lines are transition zones. The range beyond the two red lines is the error range. The value corresponding to a straight line is shown on the left and the value in black is the set-point of each subsystem, which is the average of the values corresponding to two red straight lines.

The aCAMS software was developed for European Space Agency (ESA) to examine operator performance and mental stress in the closed cabin of a spacecraft (Sauer et al., 2000). A simplified version of the aCAMS, developed by the FGAIO Group, Technische Universität Berlin, Germany (Manzey et al., 2008), was employed in our experiments. The aCAMS software has been used to design and validate AA system in Ting et al. (2010). The task of the operator is to monitor and/or control four air quality subsystems, namely O_2_ concentration, air pressure, CO_2_ concentration and temperature. All the four subsystems need to be maintained within their respective target ranges through human-machine collaboration (as shown in Figure 1A). For each subsystem, there are two control modes: automatic or manual control. Usually a subsystem is automatically controlled, but manual control has to be assumed once there is a failure of automation system. The manual control tasks may be complicated and challenging for the following reasons: (1) The target range of each subsystem is rather narrow and the control performance requirement is high; and (2) When several automation systems fail simultaneously, complex and strong coupling between individual subsystems makes it difficult for the operator to regulate such a multi-variable system manually.

#### Experimental participants

Seven healthy participants (22–24 years), A, B, C, D, E, F, and G, participated in the experiments. They were all male and healthy graduate students at East China University of Science and Technology, Shanghai. All volunteer participants were informed of the purpose and procedure of the experiment before training. Prior to formal experiments, each candidate in the participant pool was trained for over 10 h to gain sufficient experience and skills of process control operations under aCAMS task environment. Those candidates with stably (consistently) satisfactory task performance during the training were finally selected to participant in the formal experimental sessions. On the other hand, the training results indicated that the duration of the training process was sufficient for the trainees to acquire the necessary expertise on the manual control tasks.

#### Experimental procedure

In each experimental session consisting of six task-load conditions, failures in particular automated subsystems were pre-programmed. The participant was required to perform manual control until the automation systems were fixed and in normal operation. The physiological data from the participant was recorded continuously. The time response of each subsystem was displayed on a 15″ LCD monitor placed 70 cm ahead, as shown in Figure 1B. If any subsystem drifts away from the target range, the participant was required to reduce the deviation by clicking mouse on virtual buttons (switches) of O_2_ and N_2_ tank valves, CO2 scrubber, cooler, or heater. The time percentage of the output time response within the target range is used as a quantitative index of the operator performance. The interval between target and error ranges defines a transition zone, which indicates a vulnerable OFS.

The experimental method for inducing high CTL is similar to that in Ting et al. (2010). Each participant participated in two experimental sessions, which were scheduled during the same period of time (1:00–4:00 p.m.) on two consecutive days, so as to reduce the unwanted effect of circadian rhythm. After training phase, the 1st experimental session was conducted the following day. The experimental procedure in both sessions is the same. The reason for carrying out two sessions is to take into account the non-stationarity and stochasticity of the physiological and performance data recorded. Each session consisted of six task-load conditions, each of which lasted 15 min. Let discrete variable *n(k)* be the number of manually controlled subsystems at current instant *k, n(k)* = 1, 3, 4, 4, 3, and 1 correspond to each of the six consecutive task-load conditions, respectively. In other words, the task demand for the participant increased first and then decreased, both in a graded way. In the loading (or unloading) phase, the number of manually controlled subsystems was gradually increased (or reduced) stepwise. There was a short break between two consecutive conditions for participants to finish subjective ratings of perceived level of mental fatigue, effort and anxiety in the finished condition. The aim of the cyclical loading scheme (loading phase followed by an unloading phase), originally from mechanical engineering, is to examine the so-called hysteresis effect, i.e., whether and how (in what kind of pattern) the OFS recovers in the unloading phase, and the effect of the accumulated mental fatigue. Different from the experimental paradigm in (Ting et al., 2010), the condition when *n(k)* = 2 was skipped to remove the effect of the accumulated physical fatigue in an otherwise too long session. Other values of *n(k)* was the same as those in (Ting et al., 2010), i.e., *n(k)* = 1, 3, or 4. It is assumed that the level of difficulty in controlling any subsystem manually is identical.

**Figure 2:**
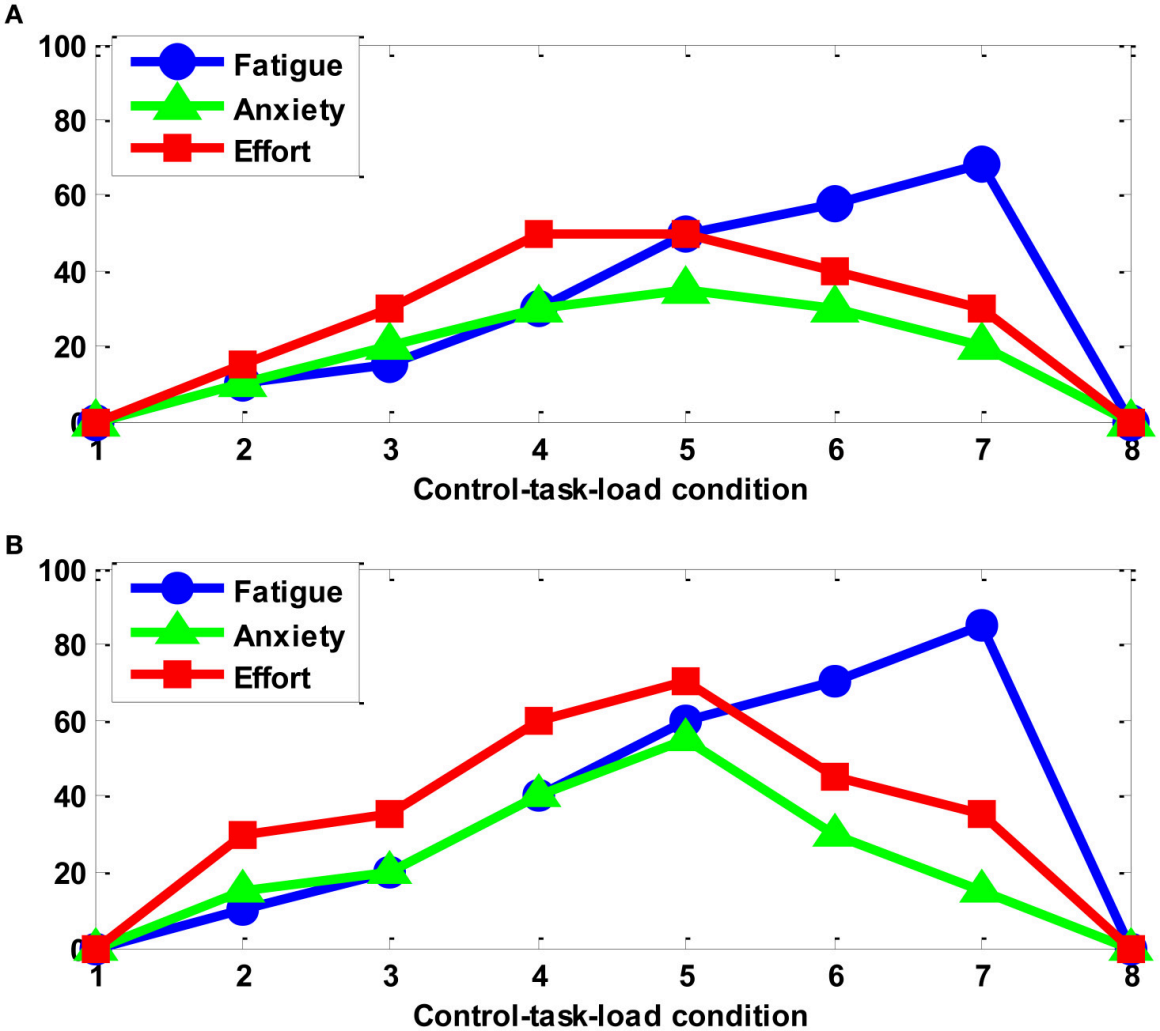
**The subjective ratings of subject A: (A)** session 1; **(B)** session 2.

Immediately after each task-load condition, the participant was required to fill in a self-assessment questionnaire handout, mainly including three rating scales of mental fatigue, anxiety, and effort (each in the range of 0–100 points). The subjective ratings for subject A are shown in Figure 2. It can be seen that all three parameters keep zero in the two baseline (unloaded) conditions #1 and #8, indicating that the mental state of the subject A is good before the start of the experiment. This is found to be the same for all other participants. By the statistical test of one-way ANOVA, the level of mental fatigue, anxiety and effort varies significantly (*p* < 0.001) across task-load conditions with different level of task difficulty. Under the designed experimental paradigm of task-load manipulation (variation), the change of the workload can be reflected to some degree by that of mental effort perceived by the participant himself. However, the self-assessment measure cannot be collected in real time by the way of questionnaire and may be too subjective for certain participants, hence we use the task performance data as another major ground truth about the objective and more real-time measure of the variations in CTL during the experiment.

### Data acquisition and pre-processing

#### Physiological data

In each experimental session, 11 channels of EEG, 1 channel of ECG, and 1 channel of electrooculogram (EOG) signals were recorded at a sampling rate of 500 Hz using Nihon Kohden® EEG device. A 3rd-order Butterworth IIR filter with a low-pass cutoff frequency of 40 Hz was used to remove the higher-frequency electromyogram (EMG) artifacts from the raw data. It should be noted that the filtered EEG and ECG data were used to extract CTL features and the EOG signal was used as a template to remove the ocular movement artifact. There were two 5-min baseline (unloaded) conditions at the beginning and end of the experimental session, respectively.

EEG electrodes included F3, F4, Fz, C3, C4, Cz, P3, P4, Pz, O1, and O2 in 10–20 international electrode placement system (as shown in Figure 1C). Before physiological data acquisition experiment, all Ag/AgCl electrodes had been cleaned and pasted using the Nihon Kodhen conductive gel. It is known that the frontal theta and parietal alpha powers are correlated to CTL variations. The correlation of the central theta and the occipital beta and gamma powers was reported in Borghini et al. (2014). Hence, all the frontal, parietal, occipital and central EEG electrodes were used in EEG signal measurement. However, it should be noted that the central channels are also known to be associated with sensory motor rhythms. Taking into this possibility, certain feature dimensionality reduction technique must be applied. The average potential of two earlobes (A1 and A2) was used as the reference. The EEG data preprocessing procedure is composed of the following steps:

1. The ocular artifact was removed from the EEG data by using the adaptive filter (see Figure 2B; He et al., 2007; Yin and Zhang, 2014):
  (1) $$v(k)=\sum_{m\;=\;1}^{M}h(m)r(k+1-m),$$
  where *M* = 3 and *h*(*m*) are the order and coefficients of filter and *r*(*k*) and *v*(*k*) are the referential EOG signal and filter output at time instant *k*, respectively. The optimal coefficients are found to minimize the cost function:
  (2) $$\varepsilon (k)=\sum_{i\;=\;M}^{k}\lambda^{k\;-\;i}e^{2}(i)$$
  where λ = 0.99 is the forgetting factor. Specifically, let $\tilde{s}\left(i\right)$ be the target EEG signal, then the artifact-free EEG signal *e*(*i*) can be computed by:
  (3) $$e(i)=\tilde{s}(i)-v(i)=\tilde{s}(i)-\sum_{m\;=\;1}^{M}h(m)r(i+1-m)$$
2. The FFT technique was used to calculate the power spectrum of each EEG epoch (10 s) with a frequency resolution of 0.1 Hz. The 10-s data epoch is selected because reliable spectral features can be derived only if the epoch is sufficiently long. Estimating CTL levels every 10 s also meets the temporal resolution (sampling rate) requirement of an online AA system. It should be noted that two consecutive EEG epochs do not overlap in order to eliminate redundancy of information content.
3. For each EEG channel, a total of 400 EEG power features (1–40 Hz with 0.1 Hz resolution) were obtained. Then, the power of five standard EEG rhythms, i.e., delta (0.5–4 Hz), theta (5–8 Hz), alpha (9–12 Hz), beta (13–32 Hz), and gamma (33–40 Hz), was computed by averaging the spectral powers in the corresponding frequency bands. As a result, the number of EEG features for each participant is 5 (No. of EEG rhythm features) × 11 (No. of channels) = 55.

ECG signal was recorded by using two electrodes placed on the top of the sternum and the bottom of the left rib cage. HR was computed based on beat intervals of two consecutive R-peaks.

Therefore, the total number of physiological features (feature dimensionality) was 55 + 1 = 56. Hence, 14 (No. of sessions) 540 (No. of data per session) × 56 (feature dimensionality) data matrices were formed. Each entry (i.e., feature) in a data matrix was normalized by subtracting the mean value and then divided by the *s.d*. of the corresponding column.

#### Performance data

In each session, the time responses of the manually controlled subsystems were recorded every second. Then, the 6 × 15 = 90 min performance data were evenly divided into 540 segments with an interval of 10 s.

Three performance indices were calculated, including the time percentage for system in error range (SIE), time percentage for system in transition zone (SIT; Ting et al., 2010), and absolute error of the system output (ASE). For each segment, The SIE at time instant *k* is defined by:

(4) $$s_{e}(k)=\frac{1}{n(k)}\sum_{i\;=\;1}^{n(k)}\frac{p_{i}}{T},$$

where *p*_*i*_ is the time duration when the subsystem *i* is out of the *error* range and *T* is the segment length (10 s). Very low *s*_*e*_(*k*) indicates the operator performance breakdown. The SIT is defined by:

(5) $$s_{t}(k)=\frac{1}{n(k)}\sum_{i\;=\;1}^{n(k)}\frac{q_{i}}{T},$$

where *q*_*i*_ denotes the time duration when the subsystem *i* is out of the *transition* zone. Low SIT indicates a vulnerable state of the operator. The ASE is defined by:

(6) $$s_{a}(k)=\frac{1}{n(k)\cdot T}\sum_{i\;=\;1}^{n(k)}\sum_{j\;=\;1}^{T}\frac{\left|d_{ij}-c_{i}\right|}{L}$$

where *d*_*ij*_ is the output of subsystem *i* at time instant *j, c*_*i*_ and *L* are the set-point and length of the error range of subsystem *i* (see Figure 1A), respectively, and *L* is used to normalize the performance of different subsystems. In general, higher AES indicates lower operator performance.

Then an overall performance measure is defined by the averaging:

(7) $$y(k)=\frac{1}{2}\min\left[s_{e}(k),s_{t}(k)\right]+\frac{1}{2}\left[1-{\tilde{s}}_{a}(k)\right].$$

Where ${\tilde{s}}_{a}\left(k\right)$ denotes the normalized *s*_*a*_(*k*), min(*s*_*e*_(*k*), *s*_*t*_(*k*)) ∈ [0, 1] indicates that the operator state would be considered risky once either *s*_*e*_(*k*) or *s*_*t*_(*k*) starts to decrease, and $\left(1-{\tilde{s}}_{a}\left(k\right)\right)$ is an extra term of performance. Lower value of *y*(*k*) ∈ [0, 1] indicates a higher CTL level.

It should be noted that the secondary task performance can also be used to label CTL classes since it is indicative of the remaining cognitive resources and the vigilance (or alertness) level of the operator when engaged with the aCAMS. However, the addition of a secondary task may interfere in the execution of primary tasks in the experiments. Alternatively, here a decrease in ASE with normal SIE and SIT is capable of indicating the performance degradation due to low level of vigilance since maintaining ASE requires the operator to pay intensive attention constantly. For this reason, we combine three performance indices in a single performance measure *y*(*k*), which can be used to distinguish low vigilance and high task demand conditions. By observing *y*(*k*), we can see that the learning effect of the 2nd session is negligible.

## Dynamic CTL classifier using physiological features

### Determination of target CTL classes

In order to evaluate the CTL classifier performance, the target CTL class *C*(*k*) at time instant *k* is determined from the performance index *y*(*k*) by:

(8) $$C(k)=\{\begin{array}{ll} 1, & 1-\sigma_{o}\leq y(k)\leq 1,\\ 2, & 1-z_{1}\sigma_{o}\leq y(k)<1-\sigma_{o},\\ 3, & 1-z_{2}\sigma_{o}\leq y(k)<1-z_{1}\sigma_{o},\\ 4, & 1-z_{3}\sigma_{o}\leq y(k)<1-z_{2}\sigma_{o},\\ 5, & \text{otherwise} \end{array}$$

where σ_*o*_ is the *s.d*. of *y*(*k*) and *z*_1_, *z*_2_ and *z*_3_ are coefficients empirically selected to discretize *y*(*k*) and given in Table 1. For each participant, the coefficient parameters are selected based on the concatenated performance data of two sessions and they are the same for both sessions of a participant. Different discretization parameters may be used due to different distribution of performance data across participants. To avoid possibly large discrepancy of the target CTL classes across participants, those discretization parameters are determined by performance data clustering for each participant separately (Yin and Zhang, 2014). Finally, CTL classes 1–5 are labeled as “very low,” “low,” “normal,” “high,” and “very high.”

**Table 1:** **The selected discretization parameter for *y*(*k*)**.

**Participant**	***z*_1_**	***z*_2_**	***z*_3_**
A	2.3	2.7	3.0
B	2.3	2.6	3.0
C	2.5	2.7	3.0
D	2.3	2.7	3.0
E	2.3	2.5	3.0
F	2.3	2.7	3.0
G	2.8	3.1	3.2

### Physiological feature smoothing and dimensionality reduction

**Figure 3:**
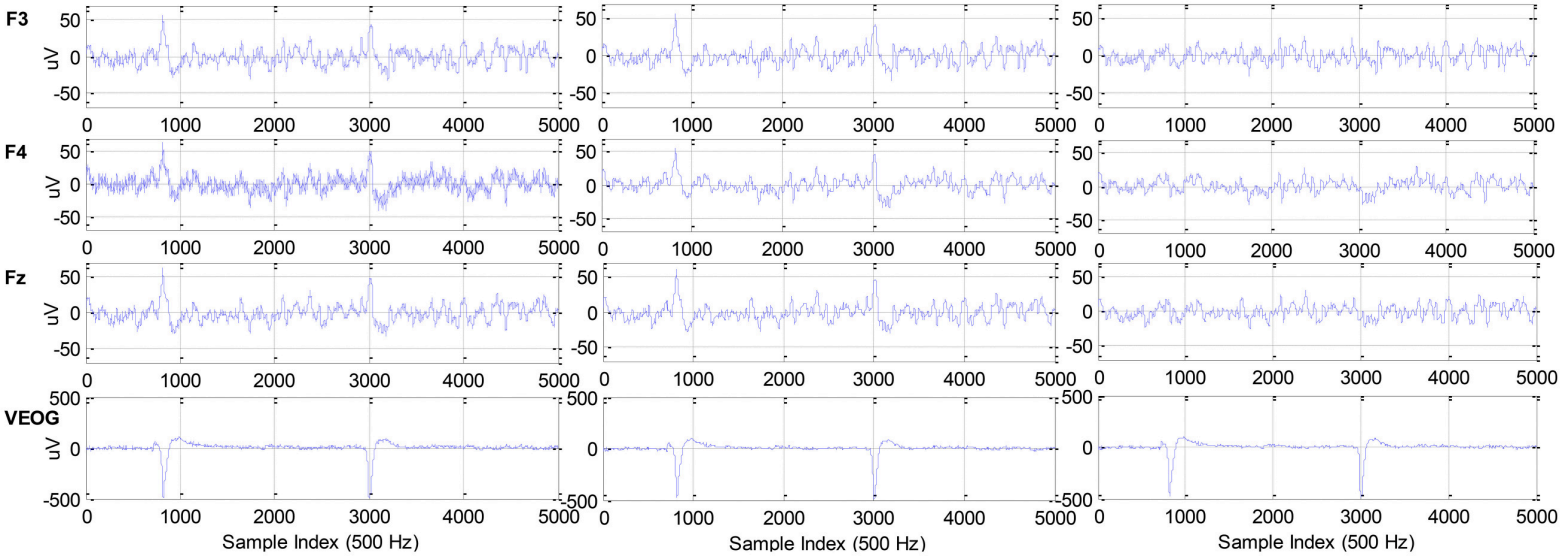
**Raw data (left)**, filtered data **(middle)**, and filtered and artifact-free data **(right)**: F3, F4, Fz of EEG, and vertical EOG from top to bottom.

The pre-filtered EEG and ECG data may still contain artifacts induced by the head motion of the participant during the experiment, thus the data was further processed by using adaptive exponential smoothing (AES) technique. In our previous work (Zhang et al., 2015), the ASE was shown to be capable of removing motion artifact with no need of the motion template while simultaneously preserving crucial information about CTL variations. Moreover, Figure 3 concretely compares the original and pre-processed physiological data.

Here the full EEG spectrum (i.e., dimensionality of feature vector is 56) is available for performing the CTL classification. However, it is known in literature that only a few EEG rhythms in certain frequency bands are primarily correlated to CTL variation and they are participant-dependent. Therefore, it is necessary to adopt certain feature reduction technique to extract the low-dimensional representation of EEG spectral features. The locality preserving projection (LPP) technique was utilized to process the EEG features in each channel in order to eliminate the components irrelevant to the CTL variations. In Zhang et al. (2015), it was demonstrated that the combination of AES and LPP methods can significantly improve the CTL classification accuracy. For each participant, AES and LPP techniques were used in the following way (as shown in Figure 3):

1. For each EEG and ECG channel, the EEG powers in five frequency bands were processed separately by:
  (9) $$\begin{array}{l} \;\varepsilon_{i}(k)=(1-f_{1})\varepsilon_{i}(k-1)+gx_{i}(k)\\ g=\{\begin{array}{ll} g_{1}, & \text{if}\left|x_{i}(k)-\varepsilon_{i}(k-1)\right|<a\sigma_{i}\\ g_{2}, & \text{if}a\sigma_{i}\leq \left|x_{i}(k)-\varepsilon_{i}(k-1)\right|<b\sigma_{i}\\ g_{3}, & \text{otherwise} \end{array} \end{array}$$
  where *x*_*i*_(*k*) and ε_*i*_(*k*) are the original and smoothed feature of the *i*-th channel (*i* = 1, 2, ···, 12), σ_*i*_ represents the *s.d*. of *x*_*i*_(*k*), and the parameters *a* = 1, *b* = 2.2, *g*_1_ = 0.2, *g*_2_ = 0.1, *g*_3_ = 0.3 (Zhang et al., 2015).
2. For ∀*i* ∈ {1, 2, ···, 11} (EEG channel), the reduced scalar feature is computed by ${x^{\prime }}_{i}=\mu^{T}{\text{x}}_{i},$ where ${\text{x}}_{i}\in R^{5}$ and **μ** is linear mapping vector derived by standard LPP (Gui et al., 2010).

**Figure 4:**
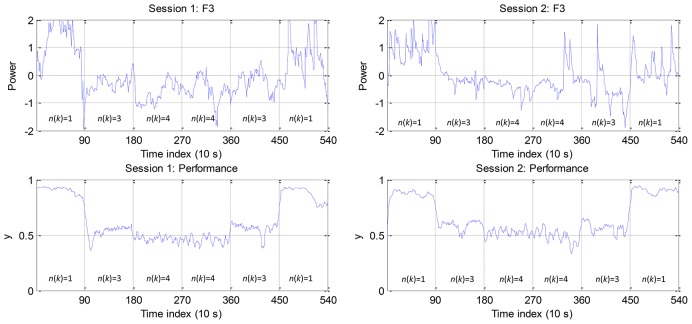
**EEG feature from the F3 channel and performance data for participant A**.

The extracted EEG F3 feature and performance data *y*(*k*) are shown in Figure 4 for participant A. It is noticed that the EEG F3 feature is positively correlated with *y*(*k*) and that the trend of feature and performance data distribution is similar between two sessions. Consequently, the CTL classifier is trained using the 1st session data and tested using the 2nd session data. Table 2 summarizes the absolute value of linear correlation coefficient (|*r*|) between 12 features and *y*(*k*). From the last column (participant-average) of the table, we can find that the EEG features from F3, Fz, C3, Cz, P3, P4, Pz, O1, and O2 are correlated to the operator performance more than other EEG channels, while HR is less correlated.

**Table 2:** **Linear correlation between physiological features and operator performance**.

	**A**	**B**	**C**	**D**	**E**	**F**	**G**	**Mean**
F3	0.8072	0.5872	0.8198	0.8088	0.7346	0.7361	0.6558	0.7356
F4	0.7667	0.6139	0.7631	0.8571	0.6781	0.6967	0.4208	0.6852
Fz	0.7745	0.6198	0.8244	0.8454	0.7475	0.7934	0.5616	0.7381
C3	0.8006	0.6500	0.8036	0.8115	0.6103	0.7825	0.7367	0.7422
C4	0.7251	0.6919	0.5832	0.8563	0.6912	0.7505	0.5595	0.6940
Cz	0.7775	0.6868	0.8119	0.8182	0.7641	0.8477	0.6300	0.7623
P3	0.8186	0.7312	0.8185	0.7786	0.6630	0.8049	0.7114	0.7609
P4	0.7980	0.7614	0.8190	0.8147	0.6943	0.7163	0.5914	0.7422
Pz	0.8474	0.7803	0.8199	0.8048	0.7199	0.8200	0.7143	0.7867
O1	0.8212	0.8649	0.8720	0.6953	0.6719	0.7799	0.5316	0.7481
O2	0.8542	0.7328	0.8382	0.7584	0.6678	0.8255	0.3468	0.7177
HR	0.4257	0.6977	0.4898	0.3774	0.0516	0.1388	0.0320	0.3161

### CTL classification via dynamic LSSVM classifier

#### Classifier structural identification

**Figure 5:**
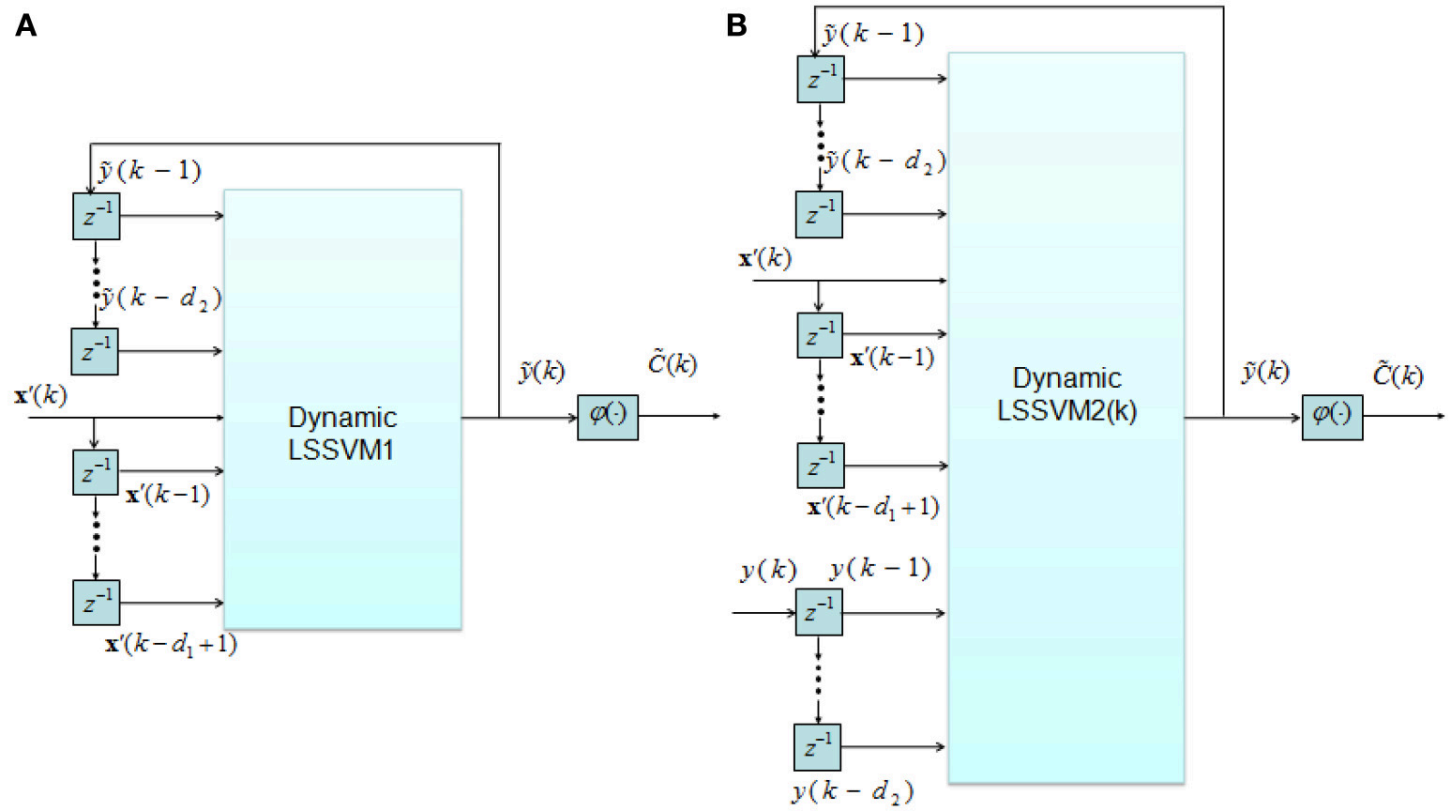
**Two structures of dynamic classifier: (A)** LSSVM1; **(B)** LSSVM2.

If both physiological and performance features are used for CTL classification, the following two NARX models can be employed (as shown in Figures 5A,B):

(10) $$\begin{array}{l} \tilde{y}(k)=f_{1}(x^{\prime }(k),x^{\prime }(k-1),\cdots ,x^{\prime }(k-d_{1}+1),\\ \;\tilde{y}(k-1),\tilde{y}(k-2),\cdots ,\tilde{y}(k-d_{2})),\\ \tilde{y}(k)=f_{2}(x^{\prime }(k),x^{\prime }(k-1),\cdots ,x^{\prime }(k-d_{1}+1), \end{array}$$

(11) $$\begin{array}{l} \tilde{y}(k-1),\tilde{y}(k-2),\cdots ,\tilde{y}(k-d_{2}),\\ y(k-1),y(k-2),\cdots ,y(k-d_{2})), \end{array}$$

where **x**′(*k*), ỹ(*k*) and *y*(*k*) denote physiological features, predicted and actual (measured) performance at time instant *k*, respectively, and the integer constants *d*_1_ and *d*_2_ are two structural parameters of the model.

The difference between two models is that the observed past performance data *y*(*k*−1), *y*(*k*−2), …, *y*(*k*−*d*_2_) appear in Equation (11). The structures of both models are shown in Figure 5.

Based on LSSVM technique for regression, Equations (10, 11) can be formulated as:

(12) $$\begin{array}{l} {\tilde{y}}_{k}=\sum_{i\;=\;d_{1}}^{l}m_{1}\alpha_{i}K_{RBF}\left(\left[{x^{\prime }}_{i},\cdots ,{x^{\prime }}_{i\;-\;d_{1}\;+\;1}\right],\left[{x^{\prime }}_{k},\cdots ,{x^{\prime }}_{k\;-\;d_{1}\;+\;1}\right]\right)\\ \;+\sum_{j\;=\;d_{2}\;+\;1}^{l}m_{2}\beta_{j}K_{Lin}\left(\left[{{\tilde{y}}^{\prime }}_{j\;-\;1},\cdots ,{{\tilde{y}}^{\prime }}_{j\;-\;d_{2}}\right],\left[{\tilde{y}}_{k\;-\;1},\cdots ,y_{k\;-\;d_{2}}\right]\right)\\ \;+\;m_{1}b_{1}+m_{2}b_{2} \end{array}$$

(13) $$\begin{array}{l} {\tilde{y}}_{k}=\sum_{i\;=\;d_{1}}^{l}w_{1}\alpha_{i}K_{RBF}\left(\left[{x^{\prime }}_{i},\cdots ,{x^{\prime }}_{i\;-\;d_{1}\;+\;1}\right],\left[{x^{\prime }}_{k},\cdots ,{x^{\prime }}_{k\;-\;d_{1}\;+\;1}\right]\right)\\ \;+\sum_{j\;=\;d_{2}\;+\;1}^{l}w_{2}\beta_{j}K_{Lin}\left(\left[{{\tilde{y}}^{\prime }}_{j\;-\;1},\cdots ,{{\tilde{y}}^{\prime }}_{j\;-\;d_{2}}\right],\left[{\tilde{y}}_{k\;-\;1},\cdots ,{\tilde{y}}_{k\;-\;d_{2}}\right]\right)\\ \;+\sum_{j\;=\;k\;-\;5}^{k\;-\;2}w_{3}\gamma_{j}(k)K_{Lin}\left(\left[{y^{\prime }}_{j\;-\;1},\cdots ,{y^{\prime }}_{j\;-\;d_{2}}\right],\left[y_{k\;-\;1},\cdots ,y_{k\;-\;d_{2}}\right]\right)\\ \;+\;w_{1}b_{1}+w_{2}b_{2}+w_{3}b_{3}(k) \end{array}$$

where $K_{RBF}\left({\text{x}}_{i},{\text{x}}_{j}\right)=\exp\left(-{\left||{\text{x}}_{i}-{\text{x}}_{j}|\right|}^{2}/\sigma^{2}\right)$ and $K_{lin}\left({\text{x}}_{i},{\text{x}}_{j}\right)={{\text{x}}_{i}}^{T}{\text{x}}_{j}$ are radial basis function (RBF) and linear kernel functions, α_*i*_, β_*j*_, γ_*j*_(*k*) ∈ *R* are the Lagrangian multipliers obtained by LSSVM training algorithm (Suykens and Vandewalle, 1999), *b*_1_, *b*_2_, *b*_3_(*k*) are biases corresponding to α_*i*_, β_*j*_, γ_*j*_(*k*), and *m*_1_ = 0.1 and *m*_2_ = 0.9 are two weights.

The model Equation (13) can be used if the history of the measured performance data *y*(*k*) is available. In Equation (13), the 1st term defines the I/O relationship between physiological features and performance, the 2nd term represents the auto-regression of the predicted performance ỹ(*k*), and the 3rd term denotes a local *d*_2_-th-order regressor of *y*(*k*). The model parameters γ_*j*_(*k*) and *b*_3_(*k*) are updated based on a small dynamic training set of only four samples (to enable fast training) by using LSSVM training algorithm. Based on the optimal training accuracy, we set *w*_1_ = 0.02, *w*_2_ = 0.18, and *w*_3_ = 0.8.

Equations (12, 13) are labeled as dynamic classifier LSSVM1 and LSSVM2, respectively. Finally, the CTL class $\tilde{C}\left(k\right)$ can be predicted by discretizing the predicted performance ỹ(*k*) according to Equation (8).

The structural parameters *d*_1_ and *d*_2_ of the dynamic classifier model must be carefully selected to guarantee the classification generalization performance. The problem is to find the best *d*_1_ and *d*_2_ to optimize the objective function:

(14) $$J(d_{1},d_{2})=\tau_{1}r_{e}(d_{1},d_{2})+\tau_{2}\frac{n_{\theta }(d_{1},d_{2})}{n_{\theta \max}(d_{1,\max},d_{2,\max})}$$

where $r_{e}=\sqrt{1/N\sum_{k=1}^{N}{\left[y\left(k\right)-ỹ\left(k\right)\right]}^{2}}$ represents Root Mean Squared Error (RMSE) between the observed (or target) and predicted performance on the training set (i.e., dataset from the 1st session), *n*_θ_ is the number of model parameters, *n*_θ max_ is the number of the model parameters with the largest possible *d*_1_ and *d*_2_,, and the two weights τ_1_ = 0.8 and τ_2_ = 0.2 are used to achieve a trade-off between the model (training) accuracy and complexity. The optimal *d*_1_ and *d*_2_ can be found such that the objective function *J* defined in Equation (14) is minimized. For this purpose, a search on a 2-D integer parameter grid [1, 15] × [1, 15] is performed, hence the number of candidate parameters is 225 (= 15 × 15). The maximal orders of the dynamical model is set to be 15 since too high order may lead to model overfitting while lower-order model is inadequate to describe the complex relationship between physiological features and performance.

**Figure 6:**
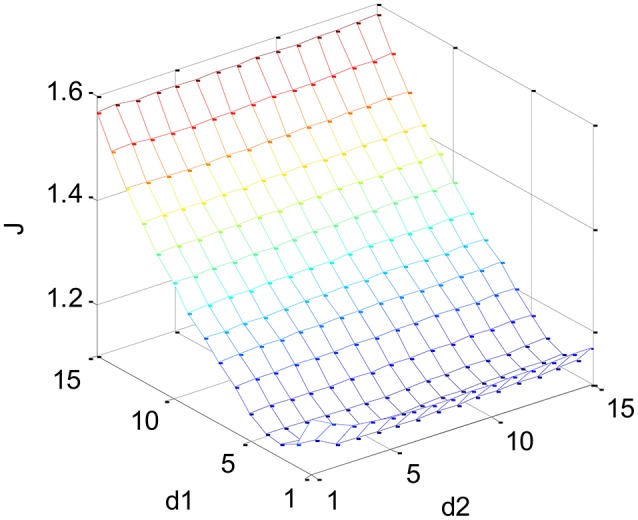
**Objective function used by classifier structural identification (participant A)**.

Figure 6 illustrates the classifier structure identification for participant A. It is shown that *d*_1_ = 3 and *d*_2_ = 5 lead to the minimum value of *J*. Table 3 gives the optimal model orders *d*_1_ and *d*_2_ for each participant. It can be observed that individual difference exists and *d*_2_ is statistically significantly larger than *d*_1_ across participants, i.e., *p* < 0.001 according to paired *t*-test with the effect size (*ES*) of −3.1302 computed by Cohen's *d*. A possible reason is that the dimensionality of the input feature of the classifier (55 here) is far greater than that of its output (1 here). As a result, too large *d*_1_ leads to a very complex model structure and thus overfitting (i.e., poor generalization capacity). The results indicated that the optimal model comprises a higher-order (i.e., 4–8) AR part (see the 2nd and 3rd term in Equation 13) and a lower-order (i.e., 1–3) moving average (MA) part. In order to compare the generalization capacity of LSSVM1 and LSSVM2 classifier, the model orders are chosen to be the same.

**Table 3:** **The classifier model order for each participant**.

**Participant**	***d*_1_**	***d*_2_**
A	3	5
B	1	5
C	2	4
D	1	5
E	1	7
F	2	6
G	2	8

#### CTL classification results

Data anlysis was carried out to evaluate the performance of two dynamic CTL classifiers. For each participant, the measured data from session 1 and 2 was used to train and test the classifier, respectively. The overall classification accuracy (*ACC*) is calculated to evaluate the classification performance.

**Figure 7:**
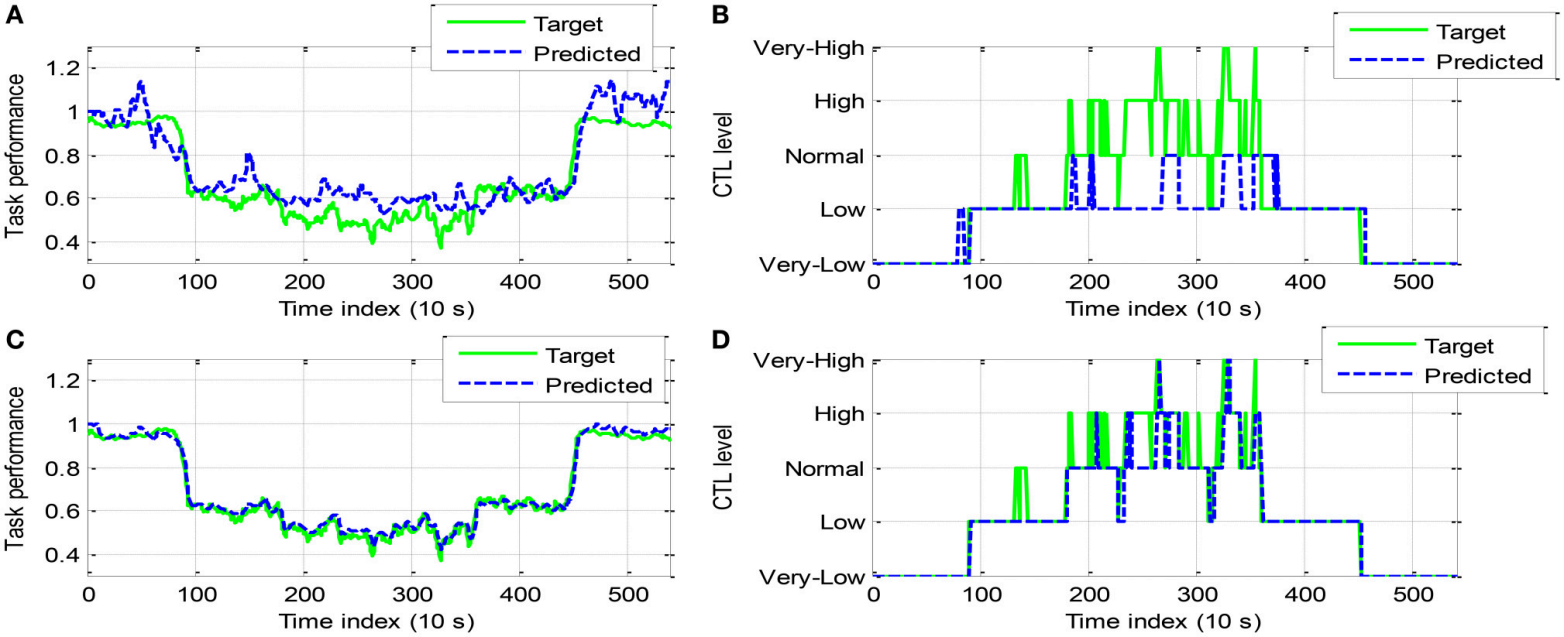
**Comparison of classification results between LSSVM1 (A,B)** and LSSVM2 **(C,D)** (participant A): **(A,C)**. Continuous output of LSSVM model; **(B,D)** Discrete CTL classes.

**Figure 8:**
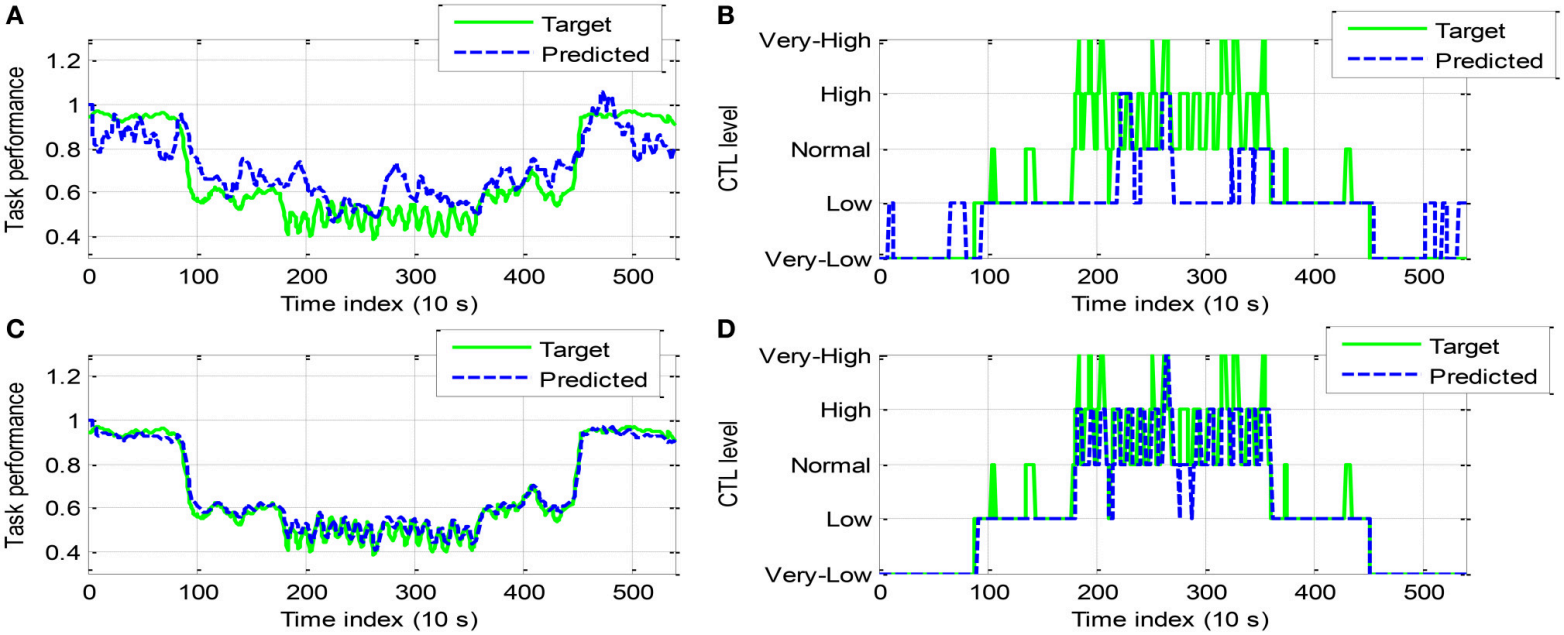
**Comparison of classification results between LSSVM1 (A,B)** and LSSVM2 **(C,D)** (participant B).

Figures 7, 8 show the classification testing results for participant A and B, respectively. The performance of the two models for CTL prediction every 10 s are compared. The same regularization parameter (100) and RBF kernel width (σ^2^ = 500) were used in the LSSVM training algorithm for two models and all participants. In general, dynamic model LSSVM2 leads to higher classification rate than LSSVM1 (i.e., 0.8500 and 0.8074 against 0.6259 and 0.5982 for participant A and B, respectively). This result suggested that the best classifier model must incorporate the measured performance in addition to the previously predicted performance ỹ (cf. Equations 11, 13). The continuous outputs (i.e., predicted performance) of LSSVM model are shown in Figures 7A,C, 8A,C, while the CTL classification results shown in Figures 7B,D, 8B,D. It is observed that the LSSVM2 model has a higher classification performance.

The classification confusion matrices are given in Tables 4, 5 for participant A and B, respectively. The class 1–5 is labeled “*very-low*,” “*low*,” “*normal*,” “*high*,” “*very-high*,” respectively. In the first column of classification confusion matrix of LSSVM1 for participant A, 168 data in *very-low* class was correctly classified, while the remaining 10 data in this class were misclassified to *low* class. Hence, the classification accuracy of *very-low* class (class 1) is 0.9438. It shows that LSSVM2 outperforms LSSVM1 in the classification accuracy of each class for both participant A and B. In particular, most data in class 3 (*normal*), 4 (*high*), and 5 (*very-high*) were misclassified by LSSVM1 (the corresponding classification rate is merely 0.0815, 0, and 0 for participant A; 0.2955, 0.0795, and 0 for participant B). Moreover, the false positive rate (FPR) and false negative rate (FNR) for binary CTL classification problem are computed in the following way.

**Table 4:** **Classification confusion matrix (Participant A)**.

**Model**	**Predicted class**	**Target class**					***ACC*_class_**	***FPR***	***FNR***
		***1***	***2***	***3***	***4***	***5***			
LSSVM1	*1*	168	1	0	0	0	0.9438	–	–
	*2*	10	162	86	51	4	0.9153	0.0562	0.0028
	*3*	0	14	8	30	6	0.0851	0.0394	0.7622
	*4*	0	0	0	0	0	0	**0**	1
	*5*	0	0	0	0	0	0	**0**	1
LSSVM2	*1*	**177**	1	0	0	0	**0.9944**	**–**	**–**
	*2*	1	**174**	17	0	0	**0.9831**	**0.0056**	**0.0028**
	*3*	0	2	**75**	50	0	**0.7979**	**0.0056**	**0.0919**
	*4*	0	0	2	**30**	7	**0.3704**	0.0045	**0.5495**
	*5*	0	0	0	1	**3**	**0.3000**	0.0019	**0.7000**

*The number of correctly and wrongly classified data is shown on the main and off diagonal, respectively. The last three columns present the classification rate of each class and the FPR and FNR for binary classification, respectively. The higher classification accuracy and lower FPR and FNR are marked in bold*.

**Table 5:** **Classification confusion matrix (Participant B)**.

**Model**	**Predicted class**	**Target class**					***ACC*_class_**	***FPR***	***FNR***
		**1**	***2***	**3**	**4**	**5**			
LSSVM1	*1*	134	6	0	0	0	0.7571	–	–
	*2*	43	156	56	53	15	0.9512	0.2429	0.0165
	*3*	0	2	26	28	3	0.2955	0.0059	0.6231
	*4*	0	0	6	7	5	0.0795	**0.0140**	0.8500
	*5*	0	0	0	0	0	0	**0**	1
LSSVM2	*1*	**177**	2	0	0	0	**1.0000**	**–**	**–**
	*2*	0	**161**	26	0	0	**0.9817**	**0**	**0.0055**
	*3*	0	1	**48**	40	1	**0.5455**	**0. 0029**	**0. 1307**
	*4*	0	0	14	**47**	19	**0.5341**	0.0326	**0.3694**
	*5*	0	0	0	1	**3**	**0.1304**	0.0019	**0.8696**

*The higher classification precision and lower FPR and FNR are marked in bold*.

In Case 1, class 1 (*very-low*) can be considered as *negative* class (“N” corresponding to *lower* level of CTL), while all other four classes considered as *positive* class (“P” corresponding to *higher* level of CTL). Similarly, the negative class can be redefined as class 1 + class 2, class 1 + class 2 + class 3, and class 1 + class 2 + class 3 + class 4, while the remaining classes redefined as positive class in Case 2, 3, and 4, respectively. The calculated FPR and FNR are listed in the last two columns of Tables 4, 5, where the 2–5th row represents the result of each classifier in Case 1–4, respectively. Take the 2nd row of Table 6 as an example, 134 and 357 instances in negative class and positive class were correctly classified by LSSVM1, but 6 data originally in the positive class and 43 data points originally in the negative class were misclassified to the opposite class. In other words, the number of false positive and false negative events predicted is 43 and 6, respectively. Then, the FPR and FNR are computed as 43/(43 + 134) = 0.2429 and 6/(6 + 357) = 0.0165, respectively. In general, the FPR and FNR of LSSVM2 are lower than LSSVM 1 in all four possible cases and the FPR are much higher than FNR for two models.

**Table 6:** **Comparison of classification testing accuracy of LSSVM classifiers and NB and KNN classifiers**.

**Participant**	**NB**	**KNN**	**LSSVM1**	**LSSVM 2**
A	0.6444	0.6870	0.6259	**0.8500**
B	0.7037	0.6333	0.5982	**0.8074**
C	0.5463	0.6759	0.4815	**0.7667**
D	0.6185	0.6482	0.4722	**0.7315**
E	0.5722	0.6074	0.5093	**0.8611**
F	0.4870	0.5426	0.5111	**0.7370**
G	0.4093	0.4926	0.4778	**0.8593**
Mean	0.5689	0.6124	0.5251	**0.8019**

*The highest classification accuracy in each row is marked in bold*.

The classification testing accuracy of two LSSVM models and classical Naive Bayes (NB) classifier and K-Nearest Neighbor (KNN) classifier (*K* = 30) is compared in Table 6. The LSSVM2 model achieved a participant-average classification accuracy of 0.8019, about 23, 19, and 28% higher than that of the NB, KNN, and LSSVM1 model, respectively. According to paired *t*-test, the LSSVM2 significantly outperforms NB (*ES* = −0.6806, *p* = 0.0019), KNN (*ES* = −0.6054, *p* = 0.0022), and LSSVM1 (*ES* = −1.0694, *p* < 0.0001). Based on the comparative results, model LSSVM2 is used to design AA controller in the next section.

## Design and simulation of adaptive human-machine system

It was found that neurophysiological data can be used to recognize the patterns of external stimuli (Speier et al., 2014; Bertrand, 2015; Comani et al., 2015). In this section, the CTL classifier is used to design an AA system that is able to realize adaptive function allocation between human operator and machine. Before online implementation of the AA system, extensive simulations based on the real data measured offline must be performed to examine the feasibility and effectiveness of adaptive task (or functional) allocation scheme. Two different adaptive task allocators, namely proportional and threshold controller and rule-based controller, are designed and tested through simulations. The primary task of the AA system, to be developed, is to use the real-time EEG and HR data *x*(*k*) to predict the performance *y* at each time step *k*.

**Figure 9:**
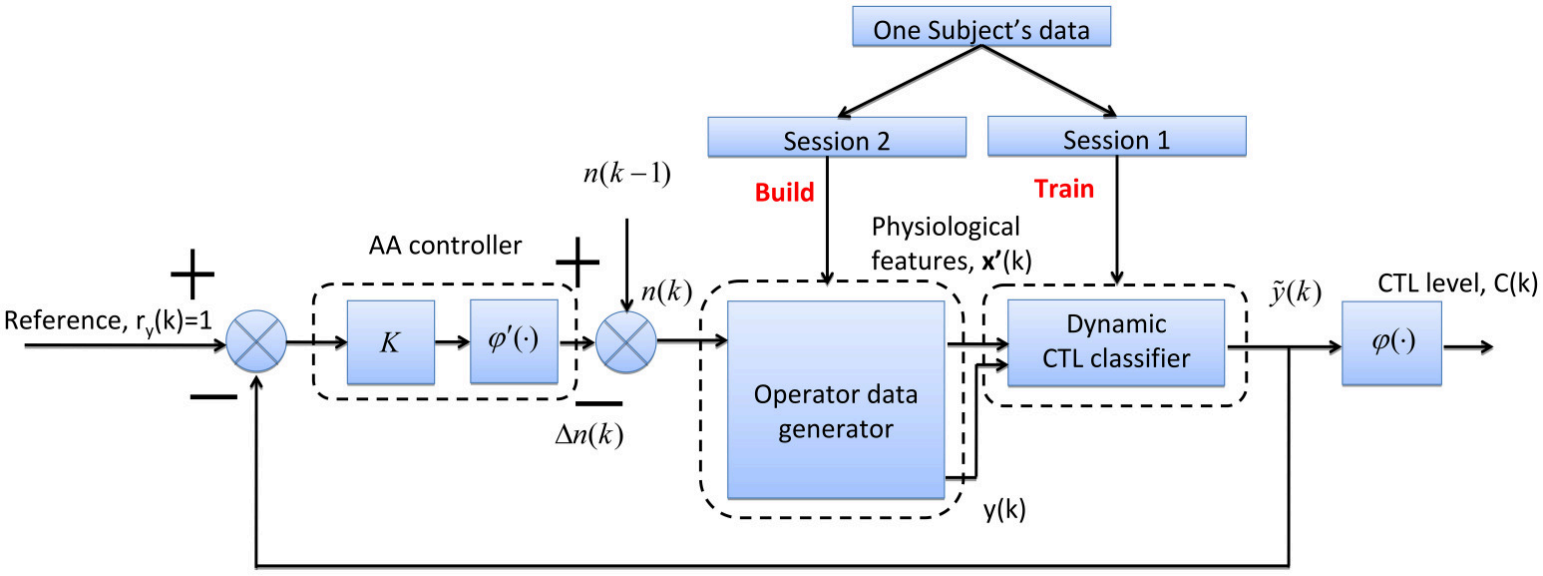
**Block diagram of AA simulation system with a proportional and threshold controller**.

A simulated AA system with proportional controller is depicted in Figure 9. The AA system with negative feedback control scheme consists of three modules, viz. data generator, dynamic CTL classifier, and controller.

1. Data generator
  The data generator is used to simulate an operator performing aCAMS tasks. For each participant, the physiological data **x**′(*k*) and performance data *y*(*k*) at time step *k* (*k* = 1, 2, ···, 90) are selected randomly among the 2nd session data. The data pairs {**x**′(*k*), *y*(*k*)} were generated every 10 s and the time duration of simulation is 10 × 90 = 900 s for each participant).
2. CTL classifier
  For each participant, LSSVM2 model is trained using the 1st session data. It should be noted that the two modules, CTL classifier and data generator, used offline experimental dataset from two different sessions, which can be easily implemented in future online human-machine system experiments.
3. Controller
  The controlled variable of simulated AA system is the operator performance ỹ(*k*) and the constant set-point (or reference step signal) *r* = 1. Based on feedback control scheme, the error signal *e*(*k*) = *r*_*y*_(*k*) − ỹ(*k*) is used as the input of AA controller. The controller is composed of a proportional gain *K* = 1 and a threshold function φ′(·) defined by:
  (15) $$\Delta n(k)=\varphi \prime \left[Ke\left(k\right)\right]=\{\begin{array}{ll} 0, & \text{ 0}\leq Ke\left(k\right)<0.2,\\ 1, & 0.2\leq Ke\left(k\right)<0.8,\\ 2, & \text{otherwise} \end{array}$$
  where Δ*n*(*k*) is incremental control action (i.e., controller output) representing the number of tasks allocated to the machine.
  Then, the number of tasks *n*(*k*) is computed by:
  (16) n(k)=n(k−1)−Δn(k)
  where *n*(*k*) ∈ {1, 3, 4} depends on **x**′(*k*) and *y*(*k*). Then *n*(*k*) is used as the input of the data generator. The physiological feature data **x**′(*k*) and performance data *y*(*k*) are selected randomly again from the task-load conditions with the same values of *n*(*k*) in the 2nd session. If *n*(*k*) = 2 is produced, the task-load condition with *n*(*k*) − 1 = 1 would be considered as *n*(*k*) = 2 is not available in our experiments.

**Figure 10:**
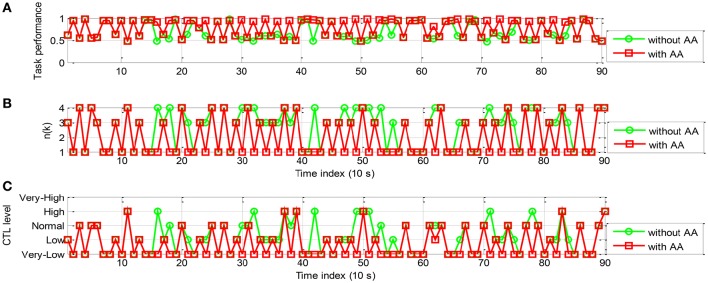
**Simulation results of AA system with the proportional and threshold controller (participant A): (A)** The operator task performance; **(B)** The number of manual control tasks allocated to the operator; **(C)** The operator CTL levels.

**Figure 11:**
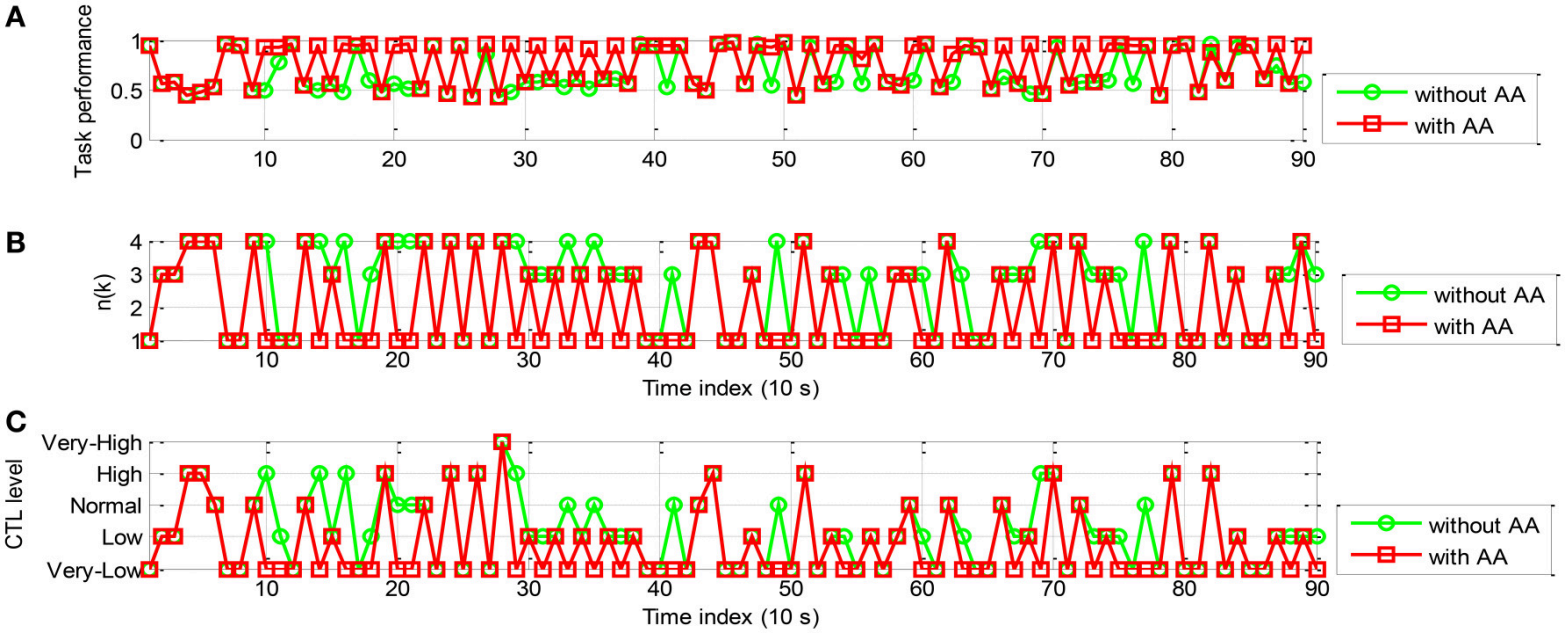
**The simulation results of AA system with the proportional and threshold controller (participant B): (A)** The operator task performance; **(B)** The number of tasks allocated to the operator; **(C)** The operator CTL levels.

The simulation results of the proportional and threshold controller are shown in Figures 10, 11 for participant A and B, respectively. In Figure 10A, with AA strategy the operator performance was enhanced at several time instants (e.g., *k* = 16, 18, 21, and 24). In Figure 10B, the control command *n*(*k*), lower than that without using AA, enables reallocation of several tasks to the machine at these time instants. Figure 10C shows the temporal variations in operator CTL level, indicating a reduction of the CTL level at those time instants. The results of participant B, as shown in Figure 11, are similar. The operator performance was improved at several time instants (e.g., *k* = 10, 11, 14, 16, and 18) due to the reduction of the CTL levels. Adaptive proportional and threshold controller was shown to be able to reduce the occurrence of the risky “*high*” and “*very-high*” CTL state of both the participant A and B.

To examine specifically how AA enhances the operator performance, the 10-run-average of the number of manually controlled subsystems, CTL level, and performance, denoted by *n*_*MEAN*_, *C*_*MEAN*_, and *y*_*MEAN*_, respectively, for each participant were given in Table 7. With AA scheme the values of *n*_*MEAN*_ and *C*_*MEAN*_ were significantly lower than those without AA according to paired *t*-test (*ES* = 1.9612, *p* < 0.001 and *ES* = 1.4022, *p* < 0.0001, respectively). Moreover, the values of *y*_*MEAN*_ were significantly improved (*ES* = −0.8514, *p* < 0.0001). The results showed that the proportional and threshold controller can not only reduce the CTL level, but also improve operator performance.

**Table 7:** **The average number of tasks allocated, CTL level, and performance with and without AA (proportional and threshold controller)**.

**Participant**	**Without AA**			**With AA**		
	****n*_*MEAN*_***	****C*_*MEAN*_***	****y*_*MEAN*_***	****n*_*MEAN*_***	****C*_*MEAN*_***	****y*_*MEAN*_***
A	2.7	2.3	0.6749	2.0	1.7	0.7849
B	2.7	2.2	0.6714	2.0	1.8	0.7761
C	2.7	2.6	0.6660	2.0	2.0	0.7606
D	2.7	2.8	0.6979	2.1	2.3	0.7704
E	2.7	2.5	0.6593	2.1	2.0	0.7508
F	2.7	2.9	0.6170	1.7	2.2	0.7261
G	2.7	1.8	0.6502	1.9	1.5	0.7468
Mean	2.7	2.4	0.6624	2.0	1.9	0.7594

**Figure 12:**
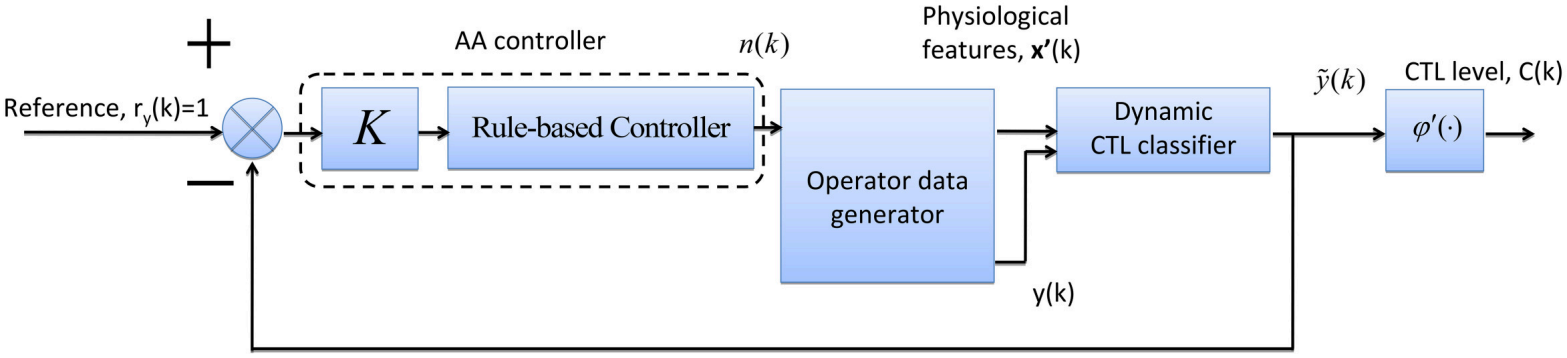
**The block diagram of AA simulation system with a rule-based controller**.

From Equation (16), we can see that the proportional and threshold controller enables task reallocation at each time step. However, certain time is required for the operator performance to recover to the set-point. Thus, *n*(*k*) needs to hold unchanged for several time steps. In Figures 10, 11, *n*(*k*) is oscillated frequently but too frequent task reallocation may heighten operator CTL level (Haarmann et al., 2009). Hence, based on the same AA simulation system structure shown in Figure 9, a rule-based controller is adopted instead to further improve human-machine system performance (see Figure 12).

Table 8 lists the three control rules used by the AA controller. The two thresholds were set to be 0.2 and 0.8 (Yang and Zhang, 2013) and gain *K* = 1. The three control rules are:

R^1^: *IF* the error *e*(*k* − 1) ∈ [0, 0.2) indicating acceptable operator performance, *THEN* the number of manual tasks at time instant *k* is unchanged, i.e., *n*(*k*) = *n*(*k* − 1).
R^2^: *IF e*(*k* − 1) ∈ [0.2, 0.8) indicating risky operator CTL level and performance (i.e., high CTL and low performance), *THEN* the level of automation is adjusted by reallocating a task to the machine, i.e., *n*(*k*) = *n*(*k* − 1) − 1, and *n*(*k*) remains unchanged at the next two time steps, i.e., *n*(*k* + 1) = *n*(*k* + 2) = *n*(*k*) (In this case, if task reallocation is triggered, the level of automation would hold constant for 30 s. This provides sufficient time for the operator to recover from high CTL state).
R^3^: *IF e*(*k* − 1) ∈ [0.8, 1] indicating operator performance breakdown and *very-high* CTL level, *THEN* two tasks are reallocated to the machine, i.e., *n*(*k*) = *n*(*k* − 1) − 2, and *n*(*k*) remains unchanged at next two time steps, i.e., *n*(*k* + 1) = *n*(*k* + 2) = *n*(*k*).

**Table 8:** **The control rules**.

*IF* 0 ≤ *Ke* (*k* − *1*) < 0.2
*n (k)* = *n (k* − *1)*,
*else if* 0.2 ≤ *Ke* (*k* − *1*) < 0.8
*n (k)* = *n (k* − *1)* − 1,
*n (k* + *1)* = *n (k)*,
*n (k* + *2)* = *n (k* + *1)*,
*else*
*n (k)* = *n (k* − *1)* − 2,
*n (k* + *1)* = *n (k)*,
*n (k* + *2)* = *n (k* + *1)*,
*end if*

**Figure 13:**
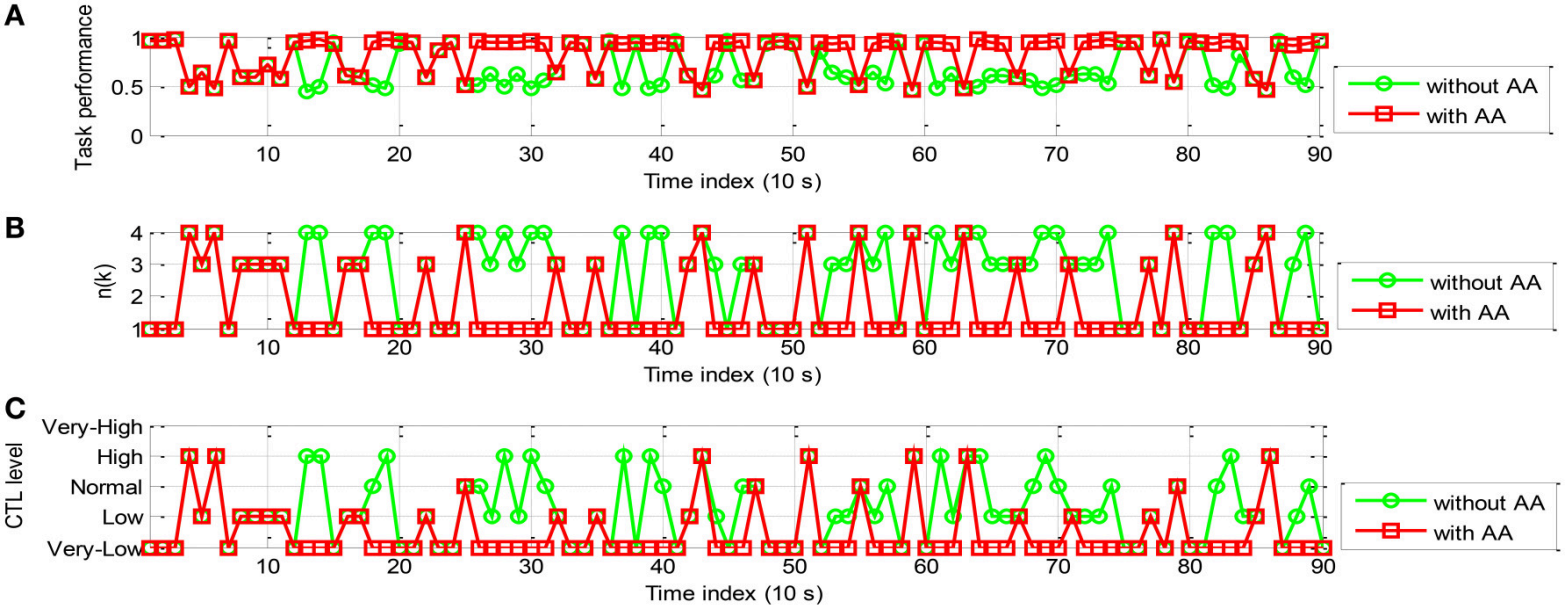
**The simulation results of AA system with the rule-based controller (participant A): (A)** The operator task performance; **(B)** The number of tasks allocated to the operator; **(C)** The operator CTL levels.

**Figure 14:**
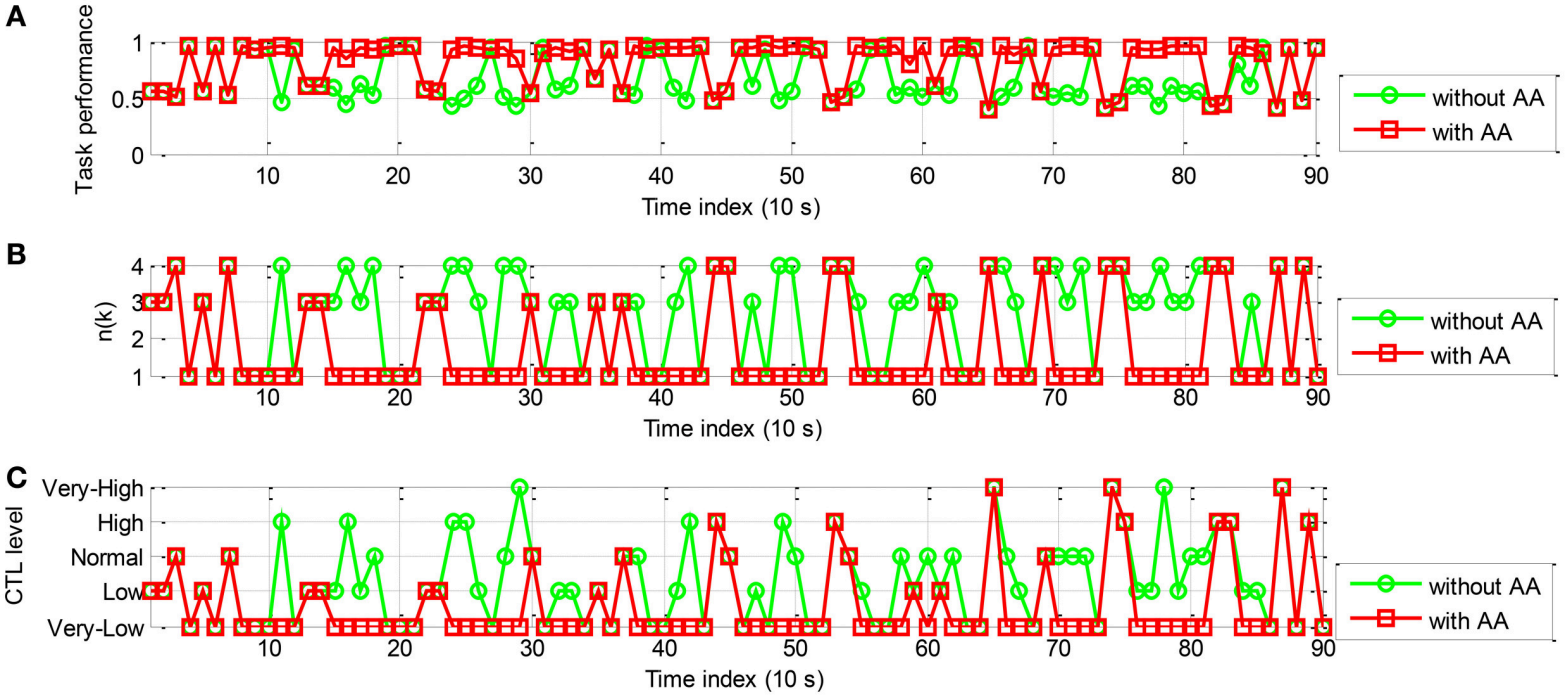
**The simulation results of AA system with the rule-based controller (participant B): (A)** The operator task performance; **(B)** The number of tasks allocated to the operator; **(C)** The operator CTL levels.

The simulation results of the rule-based controller are shown in Figures 13, 14 for participant A and B, respectively. In Figure 13A, With AA scheme the operator performance was improved at most time steps (e.g., *k* = 11, 14, 15, 16, 24, 25, 26, 28, and 29). The control action *n*(*k*) is shown in Figure 13B. The general decrease of *n*(*k*) indicated that more tasks were allocated to the machine. It is noticed that *n*(*k*) remains unchanged for at least 30 s, as a result performance breakdown was prevented. The operator CTL levels shown in Figure 13C show that most of the risky “*high*” and “*very-high*” CTL state disappears with the use of AA. The results of participant B are similar in Figure 14. Since the CTL levels have been decreased, the operator performance was improved at many time instants (e.g., *k* = 11, 15, 16, 17, 18, 23, 24, 25, 27, and 28). For participant A and B, the rule-based AA controller can effectively suppress the occurrence of risky “*high*” and “*very-high*” CTL state.

Table 9 presents *n*_*MEAN*_, *C*_*MEAN*_, and *y*_*MEAN*_ for each participant when LSSVM2 classifier and rule-based AA controller were used (10 runs of simulation). It is shown that the values of *n*_*MEAN*_ and *C*_*MEAN*_ were significantly lower than those without AA by the paired *t*-test (*ES* = 3.3814, *p* < 0.0001; *ES* = 1.7921, *p* < 0.0001, respectively). The values of *y*_*MEAN*_ were significantly improved (*ES* = −1.0622, *p* < 0.0001) when AA scheme is applied. The paired *t*-test results indicated the effectiveness of the rule-based AA scheme for enhancing the operator performance.

**Table 9:** **The average number of tasks allocated, CTL level, and performance with and without AA (rule-based controller)**.

**Participant**	**Without AA**			**With AA**		
	****n*_*MEAN*_***	****C*_*MEAN*_***	****y*_*MEAN*_***	****n*_*MEAN*_***	****C*_*MEAN*_***	****y*_*MEAN*_***
A	2.7	2.2	0.6789	1.7	1.5	0.8339
B	2.7	2.3	0.6659	1.7	1.6	0.8224
C	2.7	2.7	0.6603	1.7	1.8	0.7946
D	2.7	2.7	0.7051	1.7	1.9	0.8239
E	2.7	2.5	0.6538	1.7	1.7	0.8135
F	2.7	2.9	0.6197	1.5	2.0	0.7583
G	2.7	1.8	0.6592	1.8	1.4	0.7779
Mean	2.7	2.4	0.6633	1.7	1.7	0.8035

To compare the performance between the two AA systems, the repetitive paired *t*-test was performed to examine whether the difference in *n*_*MEAN*_, *C*_*MEAN*_, and *y*_*MEAN*_ of two AA control schemes is statistically significant. In terms of *C*_*MEAN*_, *n*_*MEAN*_ and *y*_*MEAN*_, the rule-based controller significantly outperforms the proportional and threshold controller (*ES* = 0.7411, *p* = 0.0007; *ES* = 0.8738, *p* = 0.0004; and *ES* = −0.4013, *p* < 0.0001, respectively). The average *C*_*MEAN*_ with AA is close to the “*low*” CTL (class 2) and the average *C*_*MEAN*_ in Table 8 is slightly smaller than that in Table 9 (1.7 vs. 1.9). The results demonstrated the superiority of the scheme of combining LSSVM2 classifier and rule-based controller.

## Discussion

A main contribution of this work is to propose a new NARX dynamical model architecture based on the LSSVM. We developed two types of NARX LSSVM model: dynamic LSSVM1 and LSSVM2. For the former model, the model inputs consist of EEG and ECG features at current and past time steps as well as the model output at the past time steps. For the latter model, the model inputs also incorporate the performance data at the past time steps. The LSSVM model parameters are updated based on the physiological features by means of adaptive learning/training. The basic idea and novelty of this work is the construction of a dynamic model with output (performance data) feedback. Adaptive learning algorithm is used to find the optimal parameters of the model. Therefore, we focused mainly on comparison of the classification accuracy between different model structures. The results demonstrated that the dynamic LSSVM2 model is more accurate than LSSVM1.It should be noted that the choice of adaptive learning methods may affect the CTL predictive accuracy. Hence, as an important future research direction, the best adaptive parameter learning algorithm needs to be found out for the CTL dynamic model.

The accurate and reliable estimation of CTL levels is essential to design a closed-loop HM system with human operator in the loop as it informs the controller of when and to what extent to trigger task allocation in an adaptive fashion. The dynamical LSSVM 2 is more accurate than LSSVM 1 because it incorporates the performance data at the past time steps. The NARX-LSSVM is iteratively trained with each batch of physiological sample data. The performance data is used as the model output (whose quantized value is the predicted CTL class), which can be predicted based on its historical values. A potential limitation of such a paradigm is that reliable performance data may be unavailable for some HM cooperative tasks. For instance, in driving task the deviation of the car position from the middle (centerline) of the lane can be measured continuously, while in air traffic control task real-time performance data is usually not available. In those cases where the performance data is hard or expensive to measure, the dynamical LSSVM1 model may be a more practical alternative to design the closed-loop HM systems.

On the other hand, two CTL controllers were designed to optimize the operator performance, including proportional controller and rule-based controller. Based on accurate detection of high-risk CTL level by the CTL classifier system developed, the tasks can then be dynamically reallocated between human operator and the computer. The simulation results illustrated that the number of the manually controlled subsystems can be reduced by the use of adaptive aiding strategy proposed and that both types of controller can significantly improve the operator performance and reduce the average CTL level. However, it should be noted that in a practical (or operational) adaptive system, frequent triggering of adaptive controller may bring about confusion or interference to the operators. The AA system designed does not include the functional module for detecting risky OFS due to inappropriate (or unwanted) aiding, which should be taken into consideration in the future.

## Summary and conclusion

In this paper, an adaptive human-machine system was designed and implemented by taking advantage of a dynamic CTL classifier. Fourteen sessions of experiments were performed on seven participants to measure the EEG, ECG and task performance data under simulated human-machine process control tasks. The task performance data was used to elicit five target CTL classes. The artifacts were removed from the raw EEG signals by using an adaptive filter. In total 56 features were extracted from the experimental EEG and ECG data. The AES technique was adopted to smooth the physiological feature data by removing the motion artifacts requiring no templates. The LPP technique was utilized to derive a single salient feature from each measurement channel. As a result, 12 salient markers, including 11 EEG markers highly correlated with the task performance, were extracted. Subsequently, a NARX-based LSSVM model was constructed to identify temporal variations in CTL level. Compared with static classifiers such as ANN and ANFIS, the dynamic LSSVM model takes into account the temporal correlation between current CTL state and past ones as well as current and previous physiological features. The current and past physiological features as well as previous CTL levels were used together as the input vector of the classifier model to predict the current CTL level. Essentially the NARX-based LSSVM model combines one non-linear dynamic LSSVM and two linear AR models. We examined two possible model structures and found that the CTL classification accuracy can be improved if the observed performance at previous time instants is incorporated into the classifier model. The 1st session data was used to train the classifier, while the 2nd session data was used to test its generalization (or prediction) performance. It was shown that an overall correct classification rate of about 80% was achieved for the 5-class CTL classification problem under study and the classification performance of the proposed method is robust w.r.t. the statistical non-stationarity and cross-subject variability of physiological signals. Finally, the AA system simulations using two types of controllers, namely proportional and threshold and rule-based controller, were performed for each participant. The negative feedback control system is composed of three functional modules, viz. data generator, dynamic CTL classifier and AA controller. The predicted CTL level is used to adaptively allocate tasks between human operator and computer. The obtained results demonstrated the relative superiority of the personalized AA system developed using rule-based AA controller. The subject-specific AA systems design is natural in view of the marked individual difference in physiological and task performance data. The main contribution of this work is the combination of machine learning technique with a dynamical model. Specifically, the past performance data is also used to retrain the model. Adaptive learning algorithm retrains the model iteratively and makes it straightforward to exploit the real-time operator performance information. However, the prerequisite of such a model retraining is the availability of the continuously-measured performance data. In this sense, the NARX-LSVVM model without adaptive learning function is an alternative without requiring the measurement of task performance data, which may be rather expensive or even impossible under some real-world application environments.

Finally, the limitations in current work and corresponding further work may include:

1. The number of experimental participants is relatively small, thus more participants would be required to collect more extensive experimental data in the future (Aarts et al., 2015);
2. The simulated data may not be able to account for the switch of control mode. For example, if the *n*(*k*) is increased from 1 to 4 directly, it would take some time for the operator to adapt to the high level of demanding task-load. In such a case, the operator performance likely decreases for a while at first and then increases. Unfortunately, the use of the performance data picked randomly from the simulated database does not take into account this peculiarity. Therefore, in the future online experimental work must be carried out to validate more comprehensively the practical validity and usefulness of the CTL recognition and regulation methods developed.

## Ethics statement

This study was carried out in accordance with the recommendations of Guidelines for Experiments Involving Human Subjects, Research Ethics Committee of East China University of Science and Technology, with written informed consent from all experimental participant. All participant gave written informed consent in accordance with the Declaration of Helsinki. The protocol was approved by the Research Ethics Committee of East China University of Science and Technology.

## Author contributions

JZ supervised the whole study including data analysis methods and wrote and finalized the submitted manuscript, ZY performed data analysis and wrote some parts of the first draft paper, and RW provided support to experimental work.

## Funding

This work was supported in part by the National Natural Science Foundation of China under Grant No. 61075070 and Key Grant No. 11232005.

### Conflict of interest statement

The authors declare that the research was conducted in the absence of any commercial or financial relationships that could be construed as a potential conflict of interest.
